# Explicit feedback to enhance the effect of an interim assessment: a cross-over study on learning effect and gender difference

**DOI:** 10.1007/s40037-012-0027-y

**Published:** 2012-09-27

**Authors:** Marleen Olde Bekkink, Rogier Donders, Goos N. P. van Muijen, Rob M. W. de Waal, Dirk J. Ruiter

**Affiliations:** 1 Department of Anatomy, Radboud University Nijmegen Medical Centre, PO Box 9101, 6500 HB Nijmegen, the Netherlands; 2 Department of Epidemiology, Biostatistics and Health Technology Assessment, Radboud University Nijmegen Medical Centre, Nijmegen, the Netherlands; 3 Department of Pathology, Radboud University Nijmegen Medical Centre, Nijmegen, the Netherlands

**Keywords:** Feedback, Interim assessment, Undergraduate medical education, Student’s performance, Test enhanced learning

## Abstract

In a previous study we demonstrated by a prospective controlled design that an interim assessment during an ongoing small group work (SGW) session resulted in a higher score in the course examination. As this reflects the so-called *testing effect*, which is supposed to be enhanced by feedback, we investigated whether feedback following an interim assessment would have an effect on the score of the course exam, and whether the effect is influenced by the gender of the student. During a General Pathology bachelor course all 386 (bio) medical students took an interim assessment on the topics *cell damage* (first week) and *tumour pathology* (fourth week). The intervention consisted of immediate detailed oral feedback on the content of the questions of the interim assessment by the tutor, including the rationale of the correct and incorrect answers. It concerned a prospective randomized study using a cross-over design. Outcome measures were: (1) the difference in the normalized scores (1–10) of the course examination multiple choice questions related to the two topics, (2) effect of gender, and (3) gender-specific scores on formal examination. The effect of feedback was estimated as half the difference in the outcome between the two conditions. Mixed-model analysis was used whereby the SGW group was taken as the study target. The scores of the questions on *cell damage* amounted to 7.70 (SD 1.59) in the group without and 7.78 (SD 1.39) in the group with feedback, and 6.73 (SD 1.51) and 6.77 (SD 1.60), respectively, for those on *tumour pathology*. No statistically significant effect of feedback was found: 0.02 on a scale of 1–10 (95 % CI: −0.20; 0.25). There were no significant interactions of feedback with gender. Female students scored 0.43 points higher on the formal examination in comparison with their male colleagues. No additional effect of immediate explicit feedback following an interim assessment during an SGW session in an ongoing bachelor course could be demonstrated in this prospective randomized controlled study. Gender analysis revealed a higher performance of female students on the formal examination, which could not be explained by the effect of feedback in the current study. In this particular learning environment, SGW, *explicit* feedback may have little added value to the interactive learning that includes *implicit* feedback.

## Introduction

In an ongoing effort to improve the quality of medical education and reach goals for excellence, it is important to create a stimulating learning environment for both students and teachers [1]. A relevant instrument to challenge students and teachers is assessment, as it not only drives learning but it may also help learning, the so-called ‘testing effect’ [2]. Recent work by Karpicke and Roediger has demonstrated that increased learning by testing takes place by retrieval practice, not by repeated learning [3, 4]. Underlying mechanisms include (a) focusing students on the relevant topics, (b) increasing students’ motivation, and (c) training students’ capacity to answer questions [5]. Increased learning by testing has not only been demonstrated in a laboratory environment [3, 4], but also in a more realistic setting, i.e. an ongoing course in a regular academic curriculum [6, 7]. In a previous study using a prospective controlled design, we demonstrated that an interim assessment during an ongoing bachelor course for (bio) medical students resulted in a higher score in the course examination [7]. As this study was performed during a small group work (SGW) session without explicit feedback from the interim assessment, and feedback is supposed to enhance learning by testing [3, 5, 8], we decided to embark on a follow-up study including feedback as an intervention. Since there are some indications that females are more susceptible to feedback [9], we felt it worthwhile to also study the effect of gender.

Based on a literature review, feedback can be defined as ‘Specific information about the comparison between a trainee’s observed performance as a standard, given with the intent to improve the trainee’s performance’ [10]. The purpose of feedback is to reduce discrepancies between the current understanding or performance and the desired goal [11]. It is believed that feedback provides the route by which assessment becomes a tool for teaching and learning, and it is central to the concept of formative assessment [12, 13]. Although the power of feedback is extensively emphasized in the literature, there are only a few studies that have systematically investigated the power of feedback within the classroom [13, 14]. According to a systematic review performed by Veloski et al. feedback is most effective when provided by an authoritative, credible source. These authors recommend that the effects of feedback should be studied separately from those of other concurrent interventions, such as implementation of practice guidelines or educational programmes [15]. This implies that studies on the effects of feedback in (medical) education should adhere to a well-defined description of the feedback given, and be executed with an unequivocal experimental design.

Based on these considerations we wanted to determine whether: (1) explicit feedback following an interim assessment during SGW has an effect on the formal examination score, and (2) the effect of feedback is influenced by gender. This was done by means of a prospective randomized study using a cross-over design.

## Methods

### Participants and setting

**Fig. 1:**
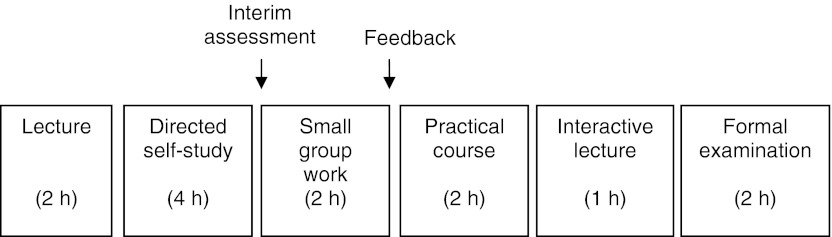
Theme structure. Time of administration of the interim assessment and feedback in relation to topic structure. The time scheduled is indicated between *brackets* for each educational component

**Fig. 2:**
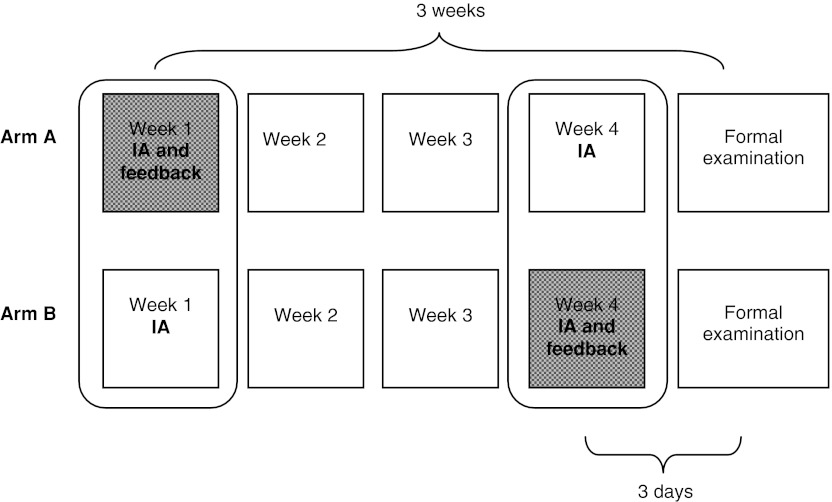
Cross-over study design. In this cross-over design, the two conditions are compared at two different moments (week 1 and week 4) as indicated in the *two vertical rectangles*. *IA* interim assessment

The study was conducted with students at the Radboud University Nijmegen Medical Centre, the Netherlands, consisting of 299 medical and 87 biomedical science students, who were all taking a second-year bachelor course in General Pathology. The female-to-male ratio of students was 3:1. A learner outcome-oriented curriculum is provided in which each course consists of four weeks including four topics (1. Principles of diagnosis and cell damage, 2. Inflammation and repair, 3. Circulatory disorders, 4. Tumour pathology). Each topic had a consistent sequence of educational activities (Fig. 1). All 386 students took an interim assessment consisting of seven multiple-choice questions (derived from a validated bank [7]) in the first (cell damage) and in the fourth (tumour pathology) week. In arm A, students received feedback on the interim assessment on cell damage; in arm B students received feedback on the interim assessment on tumour pathology (Fig. 2).

### Intervention

The intervention consisted of detailed feedback on the content of the interim assessment including the rationale of the correct and incorrect answers. This was provided orally by the tutors, who are experts in the field of general pathology. This explicit feedback was given using a standardized format, whereby all the answer options and their rationale were elucidated by the tutor. Feedback was provided according to the recommendations of Nicol and Macfarlane-Dick [16]. It took place at the end of the SGW session and lasted 10 min. In the non-feedback groups the usual discussions on cell damage or tumour pathology lasted until the end of the SGW session. Students in the control arm who asked about feedback were referred to the subsequent interactive lecture.

### Randomization

Participants from the SGW groups were randomized into two arms of equal numbers. Allocation of intervention occurred on the level of the SGW groups. A minimization procedure according to Pocock [17] and Borm [18] was used to obtain an optimal balance on the factors gender, study discipline and tutor, since these may influence learning behaviour and learning efficacy [19].

### Procedure

Students were informed about the interim assessment at the beginning of the SGW session. Tutors explained to the students immediately before the interim assessment that it was an investigation to inform the faculty on the learning outcome of students during the SGW. Participation in the interim assessment was on a voluntary basis, and students could stop taking the assessment at any time. They were assured that the result of the interim assessment would not be taken into account for determining the score of the formal course examination.

### Ethical considerations

As the study was conducted before the recently installed ethical advisory procedure of the Netherlands Association for Medical Education, information about the treatment of the students is provided. This concerns the possible risks for the students, the equitability of the selection, the guarantee of privacy and confidentiality, the procedure on informed consent, and the possible safeguards to protect vulnerable populations [20, 21]. In our opinion, participation in the interim assessments bore no possible risk to students. The assignment of the students to the intervention and control arm was at random. For the study, the examination scores were linked to the student number and the identity of the students was not disclosed. The students were adequately informed on the purpose of the interim assessment and consent was obtained. When developing the current study, the ethical principles of the World Medical Association Declaration of Helsinki were taken into account [22].

### Outcome measures

The outcome measures were: (1) The difference in normalized score of the course examination multiple-choice questions on cell damage (15 questions) and tumour pathology (15 questions). These outcome measures were presented on a scale from 1 to a maximum of 10 points; (2) The effect of gender on these differences using a subgroup analysis; and (3) The scores on the formal examination for males and females separately. As the interim assessment is intended as an educational tool, not as a predictive instrument, the results of the interim assessment were not an outcome measure of the current study.

### Statistical analysis

Linear mixed models were used in order to account for the dependence caused by clustering of the students into SGW groups; an SGW group dependent random intercept was estimated to correct for differences between SGW groups that would cause correlated residuals without this procedure. A restricted maximum likelihood estimation procedure was used and since both the number of SGW groups and the number of students within an SGW group was substantial, we used a Satterthwaite correction for the degrees of freedom. Analysis was performed according to the intention-to-treat principle. After the primary analysis, a subgroup analysis was performed according to gender.

## Results

### Participation rate

**Fig. 3:**
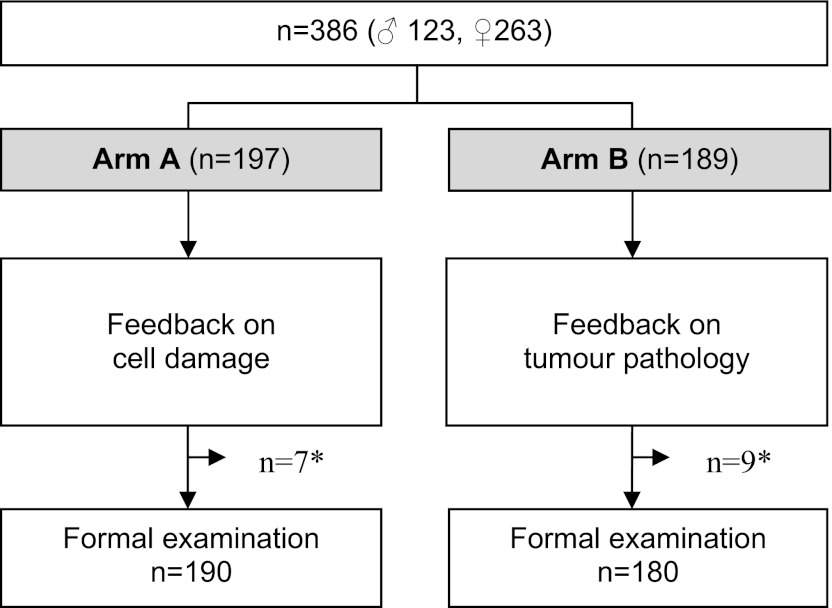
Flow chart. Study design including two arms using a cross-over design. *Asterisk* number of students excluded because they did not participate in the formal examination (*n* = 16)

Participation rate was 100 %; a total of 368 students undertook the interim assessments. Students who undertook the interim assessments but did not take the formal examination were excluded (*n* = 16) (Fig. 3).

### The effect of feedback on examination score

The scores of the questions on cell damage amounted to 7.70 (SD 1.59) in the arm without and 7.78 (SD 1.39) in the arm with feedback, and 6.73 (SD 1.51) and 6.77 (SD 1.60), respectively, for those on tumour pathology. No statistically significant effect of feedback was found: 0.02 on a scale of 1–10 (95 % CI: −0.20; 0.25) (Table 1).

**Table 1:** Outcome measures (scale 1–10) including standard deviations

Study arm	Formal examination score	Formal examination score
	Cell damage (SD)	Tumour pathology (SD)
A	*7.78 (1.39)*	6.73 (1.51)
B	7.70 (1.59)	*6.77 (1.60)*

*Italic* feedback

### Gender effect on formal examination

Female students scored 0.43 points higher in the formal examination in comparison with their male colleagues [F(1,366.262) = 11.197; *p* = 0.001].

### Gender effect on feedback

An analysis on statistical interactions between the effect of gender and feedback was performed. No statistically significant interactions of feedback with gender were found.

## Discussion

### Interpretation of the main findings

As feedback is supposed to enhance learning by testing [3, 5, 8] we decided to perform a follow-up on a previous study [7], now using feedback as an intervention. In this previous study we demonstrated that an interim assessment provided during an SGW session in an ongoing (bio) medical curriculum resulted in a higher score in the formal course exam. However, in our current prospective randomized controlled study no additional effect of immediate explicit feedback following an interim assessment in the context of an SGW session on the result of the formal course exam could be demonstrated. Females scored 0.43 points higher on the formal examination in comparison with their male colleagues. This is in line with our experience from previous years [7]. Our current study indicates that this difference in performance cannot be explained by a different gender response to feedback. Gender aspects will be discussed later.

Why were we not able to demonstrate a positive effect of feedback in the current study? To answer this question the findings will be discussed with emphasis on the educational interpretation of feedback and the methodology of the studies involved. Possible explanations could be the type and quality of feedback given, the learning environment (context) in which feedback took place, and the presence of a ceiling effect in learning. These aspects will now be discussed.

### The educational interpretation of feedback

Feedback is a process of high complexity involving different factors [23]. Its aim is to reduce discrepancies between the current understanding or performance by the student and the desired goal [11]. Also, the responses to feedback are influenced by many factors [23]. Even if feedback is appropriate and accurate, the intended message may not be recognised or understood, because the learner is not yet receptive to the message. Self-evaluation and self-regulation skills determine how feedback is received, interpreted and used by the individual student [24].

Regarding the type of feedback, Hattie and Timperley [11] propose a model of feedback to enhance learning in which they discern the task level, the process level, the self-regulation level, and the self level. The feedback given in our current study was mainly on the task level, i.e. how well the interim assessment was performed, and less on the other levels. This means that the feedback provided consisted of several components of good feedback practice as propagated by Nicol and Macfarlane-Dick [16]: It delivered high-quality information to students about their learning, encouraged teacher and peer dialogue around learning, and provided opportunities to close the gap between current and desired performance.

Regarding the quality of feedback, Wood defines helpful feedback as specific, non-judgemental, behavioural, and descriptive, and provided within a supportive educational environment close to the time of the learning experience [13]. Based on these recommendations on feedback in a (bio) medical educational setting and those by Veloski et al. [15] we consider our explicit and content-driven feedback provided by an authoritative person as specific, descriptive, and non-judgemental. It was focused on cognitive aspects and therefore not primarily behavioural. Furthermore, feedback took place at the end of the SGW session that we consider to be a supportive educational environment, as we will now discuss.

### Implicit versus explicit feedback

The learning environment in our study, i.e. the SGW session, may indeed matter, as we now elucidate. SGW is the example per excellence of active and meaningful learning [25, 26]. The power of this learning environment is determined by tutorial group functioning, the problems/tasks to be solved and tutors’ competencies [27]. The active and self-directed learner is supposed to dynamically interact with mental constructs, continuously amending and reconstructing them [25, 28]. Hereby, the learner either actively seeks feedback or detects feedback [11], which can be interpreted as implicit feedback, as it is generated by the active leaner and not provided explicitly by an external agent. Implicit feedback can also be acquired during the subsequent joint self-study, and the interactive lecture during which students can ask questions to expert tutors on remaining unsolved problems. This would mean that in the context of an optimal learning environment as the SGW session, implicit feedback is dominant in the learning process, and explicit feedback following an interim assessment has no added value in this context. Another implication is that an interim assessment in the setting of an SGW work session results in a positive effect on the formal course exam even without providing explicit feedback [7]. This discloses the interim assessment as an efficient learning instrument during an SGW, as it challenges students, fits well within the context of the learning environment, and is not time consuming.

### Comparison with the literature

To the best of our knowledge, no other prospective randomized study in which the effect of feedback following an interim assessment in the context of an SGW session has not published so far. Possible explanations for this may be that such studies are discouraged because they are strongly context dependent as suggested earlier by Van der Vleuten and Schuwirth [12], and the occurrence of publication bias because of negative results. This could be due to the fact that feedback is not necessarily a reinforcer, as it can be misunderstood, modified, or rejected [29, 30]. Few studies could be found in which the results of feedback were restricted to a subgroup of students or did not meet the a priori expectations. Murdoch-Eaton et al. [31] reported on maturational differences in undergraduate medical students’ perception about feedback. Van Mook et al. [32] were disappointed by the outcome of feedback given in the context of web-assisted assessment of professional behaviour in problem-based learning.

Regarding the effect of gender on feedback, Sinclair et al. found that female students were more susceptible to feedback than their male peers. This was related to the fact that females were keener to seek out formative feedback [9]. It is plausible that this particular behaviour may be explained by the stronger intrinsic motivation of female medical students [19] and their higher degree of mental maturation [19, 31]. This suggests that male (bio) medical students need more and/or other challenges to motivate them for learning, which should be studied further, because of their lower performance as was also demonstrated in our current study.

### Use of randomized controlled trials in medical education

Educational research grounded in qualitative paradigms greatly enriches our understanding. However, quantitative methods such as randomized controlled trials (RCTs) are still relevant in medical education research [33, 34]. This relates to the fact that they are numerous and yield important, insightful results. Before embarking on an RCT it is essential to first define the research question and conceptual framework, and then identify the most appropriate methods. For further information the reader is referred to the authoritative chapter by Norman and Eva [35]. According to Cook, RCTs will continue to play an important role in advancing our understanding in what works in medical education, for whom, and under what circumstances [33, 34]. Well-planned RCTs can test theories and explore the features and mechanisms that define effective instruction. On the other hand, one should be aware of the fact that RCTs are not appropriate for all significant and worthwhile research questions and they are susceptible, just as any other design, to the common flaws of confounding and inadequate sample size [35].

### Strengths and limitations of the current study

The study design, a prospective randomized controlled trial with minimization for gender, discipline and tutor, can be considered to be robust, since selection bias, information bias and confounding bias are highly unlikely. In addition, the cross-over design chosen prevents differences in learning environment between different groups of students.

The cross-over design in the current study has intrinsic limitations. The control arm during the second intervention may be biased by the fact that they received feedback during the first intervention; therefore, these students might be more actively searching for feedback at other instances during the course. This includes seeking feedback from their peers in the intervention arm, which may be considered to be ‘contamination’, which could lead to dilution of the effect of feedback. In addition, the time interval between the intervention and the formal course examination differed considerably, i.e. 3 weeks for the first intervention and 3 days for the second intervention. However, we did not find any difference in the effect of feedback between the first and fourth week. We did demonstrate a difference in score between the topic cell damage (first week, 7.7) and tumour pathology (fourth week, 6.7). This is in line with our experience in previous years, and it may be related to the degree of complexity of tumour pathology.

A further limitation of the current study is the strong context dependence [12], which hampers generalization of our findings. This relates to the context of feedback given during SGW and to the particular setting of our curriculum. Notwithstanding the matter of the generalization of our specific results, the current study has stimulated further thinking on the methodology and educational interpretation of feedback.

Using the current design we were not able to test the effect of the interim assessment itself. Based on the positive learning effect of an interim assessment in our previous study [7] we considered it unethical to withhold an interim assessment from the students. Therefore, all students in both conditions undertook an interim assessment. As the circumstances in the previous study were identical with exception of feedback, it is highly likely that the so-called testing effect took place.

## Conclusion

No additional effect of immediate feedback following an interim assessment during an SGW session in an ongoing bachelor course could be demonstrated in this prospective randomized controlled study. Gender analysis revealed a higher performance of female students in the formal examination, which could not be explained by the effect of feedback. In this particular learning environment, SGW, *explicit* feedback may have little added value to the interactive learning that includes *implicit* feedback.

## Essentials

- Feedback is supposed to substantially increase the positive learning effect of an interim assessment.
- The effect of explicit feedback following interim assessments during SGW sessions was studied using a prospective randomized and cross-over design.
- In this study, no additional effect of feedback on the course examination subscores could be demonstrated.
- In a SGW learning environment *explicit* feedback may have little added value to the interactive learning process that includes *implicit* feedback.
- Gender analysis revealed a higher performance of female students on the formal examination, which could not be explained by the effect of feedback.
